# Rotenone, an environmental toxin, causes abnormal methylation of the mouse brain organoid's genome and ferroptosis

**DOI:** 10.7150/ijms.74569

**Published:** 2022-07-04

**Authors:** Yongyi Huang, Xin Liu, Ya Feng, Xiaoli Nie, Qiang Liu, Xiling Du, Yuncheng Wu, Te Liu, Xiaoying Zhu

**Affiliations:** 1 School of Environmental and Chemical Engineering, Shanghai University, Shanghai 200444, China.; 2 Department of Dermatology, Yueyang Hospital of Integrated Traditional Chinese and Western Medicine, Shanghai University of Traditional Chinese Medicine, Shanghai 200437, China.; 3 Department of Neurology, Shanghai General Hospital, Shanghai Jiao Tong University School of Medicine, Shanghai 200080, China.; 4 Shanghai Geriatric Institute of Chinese Medicine, Shanghai University of Traditional Chinese Medicine, Shanghai 200031, China.; 5 School of Life Science and Technology, Tongji University, Shanghai 200092, China.

**Keywords:** Rotenone, brain organoid, genome methylation modification, ferroptosis, environmental pollution and ecotoxicity

## Abstract

More and more reports have pointed out that rotenone, as an insecticide, has high neurotoxicity and reproductive toxicity to livestock and mammals. As a highly physiological correlation system of internal organs, quasi-organs have great potential in the fields of drug toxicity and efficacy test, toxicology research, developmental biology and so on. In this study, brain organs (mBOs) derived from mouse neural stem cells were used to investigate the effects of rotenone on the physiological activity and epigenetic modification of mBOs. At the same time, Rotenone could significantly stimulate the increase of the concentration of LPO, lactic acid and hydroxyl radical in mBOs, and inhibit the expression of neuronal marker Tuj1, CHAT, PAX6 and so on. Further analysis showed that Rotenonem could induce mitochondrial damage in mBOs. The results of qPCR and Western blot showed that Rotenone could up-regulate the expressions of ferroptosis promoting protein p53, Cox2 and so on, while inhibit the expressions of negative regulatory protein of ferroptosis GPX4, FTH1, SLC7A11. In addition, the results of RRBS-Seq sequencing showed that the methylation modification at DMR level in Rotenone-treated mBOs group was significantly higher than that in Ctrl group. The results of KEGG analysis showed that compared with Ctrl, the genes with hypermethylation of promoter and Genebody in Rotenone-treated mBOs were mainly located in the Neuro active ligand-receptor interaction signal transduction pathway. In summary, rotenone can significantly lead to abnormal methylation of mouse brain organs, and lead to the loss of normal physiological function of neurons by inducing ferroptosis.

## Introduction

Rotenone is a kind of organic matter, the molecular formula C_23_H_22_O_6_, exists in the leguminous ichthyoteng plant roots, at the same time, in some Chinese herbs such as sunflower seeds, kudalwood seeds, kunming chicken spatoba root also contains.When exposed to air, it oxidizes and precipitates dehydrorotenone, which is toxic to insects 1. Rotenone was originally used as an insecticide. When it entered the insect body, it would inhibit the mitochondrial electronic respiratory chain of insect somatic cells, leading to respiratory disorders such as dyspnea and convulsion, slow action, paralysis and death 2. Rotenone is also used as a snail killer and to catch ornamental fish 3. Rotenone was originally thought to be very safe for humans and animals, in addition to being toxic to insects and aquatic animals. However, more and more studies have reported that rotenone has high toxicity to domestic animals, fish, silkworm and mice, especially neurotoxicity and reproductive toxicity 4-8. Our previous study found that rotenone enhances the transcriptional activity of p53 gene and induces the apoptosis of dopamine neurons by targeting the regulation of Sirt1 and histone H3K9 acetylation modification 9. At the same time, the frequent use of rotenone as an insecticide and snail killer in agriculture and aquaculture has greatly increased the probability of soil and underground water contamination with rotenone 10. Therefore, rotenone has become a potential environmental ecotoxin, which seriously threatens the ecological health of the environment.

Organoids are designed to induce stem cell populations to differentiate into certain tissues and organs *in vivo* through a 3D culture system. Organoids possess similar spatial structures to their corresponding organs and are able to reproduce some functions of the corresponding organs, thus providing a highly physiologically relevant system 11-13. Organoid cells can be generated from tissue samples containing adult stem cells, single adult stem cells, or through directed induction of pluripotent stem cells 12, 13. Organoid is a highly informative complement to 2D cell culture methods and animal model systems 12, 13. As a model biological tool, organoids have great potential in drug toxicity and efficacy test, toxicology research, developmental biology and other fields 14-17. So far, organoid cultures have been used in a variety of tissues, including the gut 18, 19, liver 14, 20-22, pancreas 20, kidney 23, prostate gland 24, lung 25, 26, Optic cup 27 as also as the brain 28, 29. However, there are few researches on the application of Organoids in environmental pollution and ecotoxicology.

DNA methylation is a normal and common modification in eukaryotic cells and the main epigenetic form of gene expression regulation in mammals 30-32. The so-called DNA methylation refers to the covalent bonding of a methyl group at the fifth carbon atom of the cytosine of CpG dinucleotide in the genome under the action of DNA methylation transferase 30-32. A large number of studies have shown that DNA methylation can control gene expression by altering chromatin structure, DNA conformation, DNA stability and the interaction between DNA and protein 30-32. DNA methylation typically occurs in the CpG island region of a gene's promoter, or 5' non-coding region and the first exon region 30-32. In general, DNA methylation blocks the transcription of genes and reduces their expression levels. Dana M Freeman et al. found that non-germline allele-specific DNA methylation seem conserved between mouse and human genomes, which supported the notion that allele-specific DNA methylation are sensitive to environmental factors such as rotenone and may alter the risk of neurological disease later in life by disrupting neuronal development 33. Gustavo Scola et al. reported that rotenone decreased mitochondrial complex I activity and ATP production via increasing levels of 5-methylcytosine and hydroxymethylcytosine, suggesting a possible association between complex I dysfunction and DNA alterations 34. Although the above reports have revealed that rotenone is closely related to the epigenetic regulation of mammalian genomic DNA methylation modification, in-depth mechanism studies have not been reported.

In this study, we intend to construct mouse brain organs (mBOs) using mouse neural stem cells *in vitro* and take them as the research object. We use high-throughput DNA methylation sequencing technology to conduct whole-genome scanning of mouse brain organs with or without rotenone treatment, in an attempt to clarify the epigenetic toxicity of rotenone. And the molecular biological mechanism of ferroptosis induced by it.

## Materials and Methods

### Murine brain organoids (mBOs) established and Rotenone treated

Reference to existing literature 35, 36, briefly, NE-4C cell lines were adhered to culture until overgrown, digested by Accutase, and cell deposits were collected by centrifugation. The cell precipitate was suspended with 0.5 ml ice pre-cooled base medium, and 0.5 ml ice pre-cooled Matrigel was added, fully blown and mixed, and the cell suspension drops were added to the non-adhesive cell culture dish, with each drop of cell suspension controlled at about 0.1 mL. The cells were placed in a 37 °C cell culture box with 5% CO_2_ and incubated for 15 minutes. Then, 4 ml of preheated basal medium at 37 °C was added and placed in a cell culture box at 37 °C with 5% CO_2_. Two days later, the original medium was abandoned and 4 ml induction medium 1# was added again to continue culture. Two days later, the original medium was abandoned, and 4 ml induction medium 2# was added to continue culture. Then, the fresh medium 2# was changed every 2 days. At about 10 days, the formation of distinct cell clones was visible. Basal medium (Advanced DMEM-F12 83ml + FBS 15ml + Penicillin-streptomycin 1ml + L-glutamine 1ml). Induction medium 1# (Advanced DMEM-F12 80 ml + FBS 15 ml + Penicillin-streptomycin 1 ml + L-glutamine 1 ml + B27 supplement 2 ml + N2 supplement 1 ml + Activin A 10ng/ml + bFGF 10 ng/ml + EGF 10 ng/ml + RA 10 ng/ml + vEGF 10 ng/ml + ASCORBIC ACID 50 ng/ml). Induction medium 2# (Advanced DMEM-F12 75 ml + FBS 15 ml + Penicillin-streptomycin 1 ml + L-glutamine 1 ml + B27 supplement 2 ml + N2 supplement 1 ml + Activin A 10 ng/ml + bFGF 10 ng/ml + EGF 10 ng/ml + vEGF 10 ng/ml + ASCORBIC ACID 50 ng/ml + R3-IGF-1 10 ng/ml + HYDROCORTISONE 10 ng/ml + HEPARIN 10 ng/ml). According to previous studies 9, the concentration of rotenone treated cells was briefly 1.0 μM.

### Transmission electron microscopy (TEM)

Samples were fixed and embedded as previously reported 37. Briefly, tissue samples were fixed in 1% glutaraldehyde (Sigma-Aldrich, St. Louis, USA) for 4 h and then in 1% osmium acid (Sigma-Aldrich) for 1 h. After dehydration in acetone, they were embedded in resin 12 (Ted Pella, USA). Ultrathin sections (cross-section thickness = 70 nm) were mounted on a copper mesh, stained with 1% uranyl acetate (Sigma-Aldrich) and 1% lead citrate (Sigma-Aldrich), and observed and photographed under a JEM-1230 transmission electron microscope (JEOL, Japan).

### RNA extraction and qPCR

Briefly 37, RNA extraction was performed using TRIzol (Invitrogen Life Technologies, Carlsbad, CA, USA), according to manufacturer instructions. After exposure, each group of 6000 nematodes was placed in 1.5 mL Eppendorf tubes and washed three times with PBS. TRIzol reagent (1 mL; Invitrogen) was then added, and a tissue lyser was used (50 Hz and 5 min) to obtain tissue homogenates. Total RNA was treated with DNase I (Sigma-Aldrich), and RNA concentration was determined using a NanoDrop 1000 spectrophotometer (Thermo Scientific, USA) by measuring UV absorbance at 260 nm. Purity was assessed by determining the absorbance ratio at 260/280 nm. Total RNA (1-2 μg) samples were then subjected to reverse transcription for cDNA synthesis with MMLV reverse transcriptase (Promega). Briefly, 0.5 μg oligo dT_18_ was added to each tube, followed by incubation at 70 °C for 5 min to melt secondary structures within the template; the mixture was then immediately kept on ice. Reverse transcription was then performed using a mixture comprising 5 μL of MMLV 5× reaction buffer, 1.25 μL of 10 mM dNTP mixture, 25 U of ribonuclease inhibitor (Sigma), 200 U of MMLV (Promega), and RNase-free water to adjust the final volume to 25 μL. cDNA synthesis was achieved by incubation at 42 °C for 60 min and 95 °C for 5 min, and cDNA was then stored at -20 °C. qPCR was performed on a RealPlex4 real-time PCR detection system (Eppendorf Co. LTD, Hamburg, Germany) with SYBR Green Real-Time PCR Master Mix (Toyobo (Shanghai) Biotech Co., Ltd.). qPCR included 40 cycles of initial denaturation at 95 °C for 15 s, annealing at 58 °C for 30 s, and extension at 72 °C for 42 s. The 2^-ΔΔCt^ method was applied to measured relative gene expression levels, wherein ΔCt = Ct_genes-Ct_18S rRNA and ΔΔCt = ΔCt_Rotenone_groups-ΔCt_PBS_group. mRNA expression level was corrected based on 18S rRNA expression level. Finally, the results of qPCR assay were presented in the form of heatmaps.

### MTT assay

Briefly, 2×10^3^ cells were inoculated into 96-well cell culture plates. After 24 h, cells in each group were added with 10 ul MTT solution (sigma-aldrich, st. Louis, USA) and incubated at 37 °C for 3 h. The culture medium was discarded, 150 µl DMSO (Sigma-Aldrich, St. Louis, USA) was added to each well, and shaken for 15s. The cell culture plate was placed in a microplate reader, and the absorbance value at the wave length of 450nm was recorded. Cell proliferation inhibition rate (%) was calculated as: (1-OD value of experimental cells -blank/ OD value of control cells -blank)×100%.

### Iron (Fe2+) assay

Intracellular ferrous iron level was determined using the iron assay kit (Abcam, ab83366) according to the manufacturer's instructions 38.

### Lipid peroxidation (LPO) assay

The relative MDA concentration in cell or tumor lysates was assessed using a Lipid Peroxidation (MDA) Assay Kit (Abcam, #ab118970) according to the manufacturer's instructions. Briefly 38, MDA in the sample reacts with thiobarbituric acid (TBA) to generate a MDA-TBA adduct. The MDA-TBA adduct can be quantified colorimetrically (OD = 532 nm). C11-BODIPY dye (Thermo Fisher Scientific) was used to detect lipid peroxidation in cells. Oxidation of the polyunsaturated butadienyl portion of the dye results in a shift of the fluorescence emission peak from ~590 to ~510 nm.

### Glutathione (GSH/GSSG) assay

The relative GSH concentration in cells was assessed using a GSH/GSSG Ratio Detection Assay Kit (Abcam, #ab205811) according to the manufacturer's instructions. Briefly 38, whole cell was diluted to 1:80 for GSH analysis, serial dilution of GSH and GSSG stock standards were prepared as standards. A one-step fluorimetric reaction of samples with respective assay buffer and probes was incubated for 30 min. The yellow product (5-thio-2-nitrobenzoic acid) was measured spectrophotometrically at 412 nm.

### Adenosine triphosphate (ATP) assay

The adenosine triphosphate (ATP) assay was performed according to the manufacturer's protocol from the Enhanced ATP Assay Kit (Beyotime, Shanghai, China) 39. Briefly, 200 μL of the sample lysate was added to 1×10^6^ cells from murine brain organoids and thoroughly mixed by pipetting up and down. After centrifuging at 12,000 g for 5 minutes at 4 °C, the supernatant was collected. At the same time, the ATP standard solutions were set up. The ATP standard solutions were adjusted to the following concentrations: 0.01, 0.03, 0.1, 0.3, 1, 3, and 10 μM, respectively, and were tested with samples together. Fresh testing solutions were prepared as required by the kit's protocol. ATP testing solution (100 μL) was added to each of the testing wells and standard wells, and they were incubated at room temperature for 5 minutes. Then, 20 μL of the test sample or standard solution was added to the wells and quickly mixed. After 5 seconds at room temperature, the relative light unit (RLU) values were measured using a luminometer.

### Superoxide dismutase (SOD) assay

The superoxide dismutase (SOD) assay was performed according to the manufacturer's protocol from the SOD Activity Assay Kit (Beyotime, Shanghai, China) 39. Briefly, 200 μL of the sample lysate was added to 1×10^6^ cells/mL and was thoroughly mixed by pipetting up and down. The mixture was then centrifuged at 12,000 g for 5 minutes at 4 °C, and the supernatant was collected. The WST-8 enzyme working solution was prepared by thoroughly mixing 151 μL of SOD assay buffer, 8 μL of WST-8, and 1 μL of enzyme solution. At the same time, a concentration gradient of SOD standard solutions was set up. The SOD standard solutions were diluted to 100U/mL, 50U/mL, 20U/mL, 10U/mL, 5U/mL, 2U/mL, and 1U/mL, respectively, and were tested simultaneously with the samples. Twenty microlitres of the supernatant of the cell lysis and standard solutions were collected and added to 160 μL of freshly prepared WST-8 enzyme working solution and 20 μL of reaction initiation solution, respectively. After thorough mixing, the samples were incubated at 37 °C for 30 minutes. The absorbance was measured at 450 nm.

### Western blot

Briefly, the total proteins from each group of cells were electrophoresed using 12% SDS-PAGE denaturing gel, and then transferred to a PVDF membrane (Millipore). After sealing and washing, the PVDF membrane was incubated with primary antibodies at 37 °C and allowed to react for 45 min. After thoroughly washing, the PVDF membrane was incubated with second antibodies for a reaction time of 45 min at 37 °C. We washed the PVDF membrane with TBST four times at room temperature for 14 min each time. We then used enhanced chemiluminescence ECL Kit (Pierce Biotechnology) and exposed and developed the film (Sigma-Aldrich Chemical).

### Hematoxylin and eosin (H&E) staining

In brief, all of the fresh tissues were immersed in 4% paraformaldehyde (Sigma Aldrich, St. Louis, USA) at room temperature for 30 min, dehydrated through a graded series of ethanol, embedded in paraffin, sectioned at 6 μm, and slides soaked in xylene for dewaxing. Histologic sections were stained with hematoxylin-eosin (H&E, Sigma Aldrich, St. Louis, USA) and finally coated with xylene (Sigma Aldrich, St. Louis, USA) and neutral resin (Sigma Aldrich, St. Louis, USA).

### Immunofluorescence staining

In brief, all of the fresh tissues were soaked at room temperature and fixed in 4% paraformaldehyde (Sigma-Aldrich, St. Louis, USA) for 30 min. We performed ethanol-gradient dehydration, paraffin embedding, tissue sectioning at a thickness of 6 μm), and dewaxing in xylene. The tissue sections were sealed at 37 °C for 30 min with immunohistochemical blocking solution (Beyotime Biotechnology Co., Ltd., Zhejiang, China). We discarded the blocking solution and added immunohistochemical cleaning solution (Beyotime Biotechnology Co., Ltd., Zhejiang, China) to rinse sections three times at room temperature for 5 min each. Primary antibodies were added and incubated at 37 °C for 45 min. We discarded the antibodies and added immunohistochemical cleaning solution (Beyotime Biotechnology Co., Ltd., Zhejiang, China) to rinse three times at room temperature for 5 min each. Then, the secondary antibodies were added and we incubated sections at 37 °C for 45 min. After discarding the antibodies, we added the immunized histochemical cleaning solution (Beyotime Biotechnology Co., Ltd., Zhejiang, China) and rinsed at room temperature for 5 min 3 times. Finally, an immunofluorescence sealing solution (Sigma-Aldrich, St. Louis, USA) was added to seal the tablets.

### High throughput sequencing of genomic DNA methylation (RRBS-Seq)

According to the previous studies 40, 41, birefly, transposome complex was first generated by incubating 2.5 μl of 10 μM annealed adaptors with 2.5 μl 100% glycerol and 5 μl Ez-Tn5 transposase (Epicentre, Illumina) for 30 min at 25 °C. Cells were lysed by proteinase K treatment for 40 min at 37 °C. The genomic DNA was purified by AMPure XP magnetic beads (Beckman Coulter). The purified DNA (∼ 0.5 ng, spiked with 5 pg of unmethylated lambda DNA) was then incubated with 4 μl Nextera HMW Buffer (Epicentre-Illumina), 16 μl nuclease-free water (Ambion), and 4 μl prepared Tn5mC transposome complex for 12 min at 55 °C followed by purification using 36 μl (1.8×) Agencourt AMPure XP magnetic beads; and the DNA was eluted in 14 μl EB buffer (Qiagen). An extension step was then performed by adding 2 μl of 10× Thermopol reaction buffer (New England Biolabs), 2 μl 10 mM 5mC dNTP Mix (Zymo Research), 1 μl of Bst DNA polymerase large fragment (New England Biolabs) to each reaction mixture and incubated for 20 min at 65 °C. Each reaction was spiked with 200 ng of sonicated unmethylated lambda DNA (200-400 bp) (Takara) and then subject to bisulfite conversion using a Zymo EZ DNA Methylation Kit (Zymo Research) following manufacturer's protocols involving a 14 h 50 °C incubation at dark and 22 μl (2 × 11 μl) elution. The purified DNA was then amplified using 25 μl Kapa 2G robust hot start ready mix (Kapa Biosystems), 1 μl 50× Nextera primer cocktail (Illumina - compatible) and 1 μl barcoded Illumina-compatible adaptor 2 (8-bp index) on a thermocycler with the following parameters: 1 min at 95 °C followed by 10-15 cycles of 20 s at 95 °C, 30 s at 60 °C, 45 s at 72 °C. The prepared libraries were analyzed by Agilent 2100 Bioanalyzer (Agilent Technologies) and quantified by Quantitative PCR (qPCR) and then used for cluster generation and pair-end sequencing with 90 bp reads (PE90) on Illumina Hiseq 2000 (Illumina). The sequencing test was completed by sz-acegen (BGI, Shenzhen, China) and Biomarker (Beijing, China).

### Statistical analysis

Each experiment was performed as least three times, and values are reported as mean ± standard error, where applicable. Differences were evaluated with Student's *t*-test. p < 0.05 indicated statistical significance.

## Results

### Rotenone promotes oxidative stress damage of mBOs

**Figure 1:**
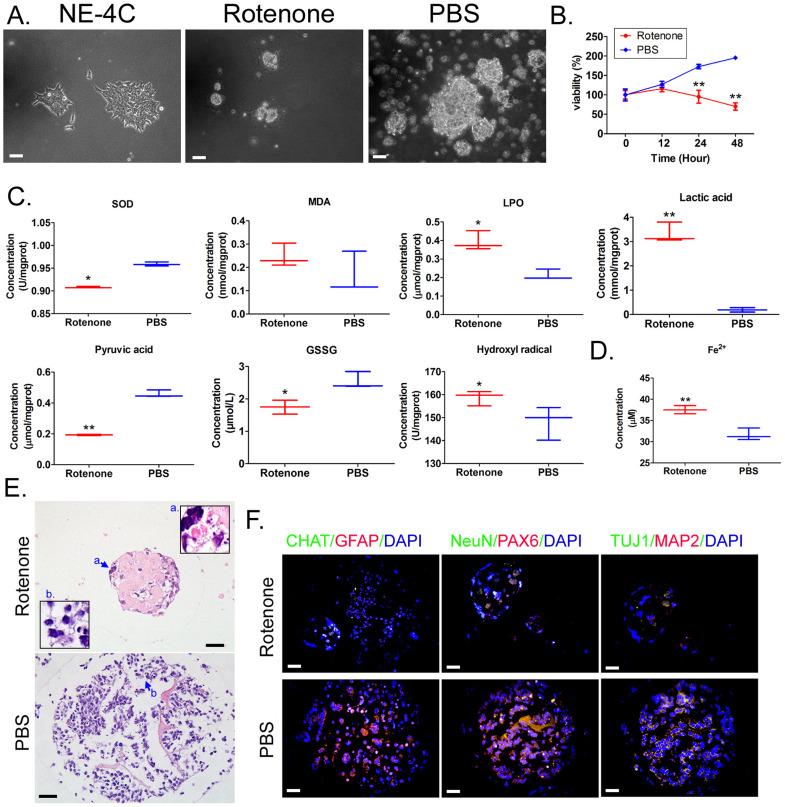
** Rotenone promotes oxidative stress injury in mBOs. A.** Morphology of NE-4C cell lines before and after induction under light microscope. The magnification is 200x. Scale bar = 30 µm. **B.** MTT results suggested that Rotenone suppressed the proliferation of NE-4C-derived mBOs *in vitro*. **p < 0.01 vs PBS; Student's t-test. **C.** Oxidative stress test results showed that Rotenone induced increased lipid peroxides and decreased antioxidant capacity of NE-4C-derived mBOs.**p<0.01 vs PBS; *p<0.05 vs PBS; student's t-test. **D.** Rotenone induced a significant increase in Fe2+ concentration in NE-4C-derived mBOs.**p<0.01 vs PBS; Student's t-test. **E.** H&E staining indicated that Rotenone damaged the morphology of neurons in NE-4C-derived mBOs. The magnification is 200x. Scale bar = 30 µm. **F.** Immunofluorescence staining indicated that Rotenone inhibited the expression of neuronal markers in NE-4C-derived mBOs. The magnification is 200x. Scale bar = 30 µm.

Mouse neural stem cells NE-4C were used to construct mBOs *in vitro* suspension culture.Under light microscope, NE-4c adherent growth showed clonelike morphology (Figure 1A). After the treatment of mBOs with rotenone, the mBOs in the control group (PBS) had significant spherical clon-like structures (Figure 1A), while mBOs in the Rotenonem group had significantly smaller cloned spheres and even a large number of dead cells floating on the culture medium (Figure 1A). MTT results showed that the proliferation inhibition rate of mBOs increased significantly with the increase of Rotenonem treatment time (Figure 1B). Biochemical tests showed that Rotenone treatment significantly increased LPO, lactate and hydroxyl free radicals in mBOs (Figure 1C), but significantly decreased SOD, GSSG and pyruvate concentrations compared with the control group (Figure 1C). Meanwhile, Rotenone treatment significantly increased Fe2+ in mBOs (Figure 1C). H&E staining results showed that multiple neuron-like cells with large and bulging nuclei and synapses with multiple cells could be seen in mBOs clone spheres of the control group (Figure 1E). However, in the rotenone-treated group, most neuronal cells atrophied and the nuclear morphology was unclear, suggesting cell death (Figure 1E). Immunofluorescence staining results showed that after Rotenone treated mBOs, the positive levels of neuron markers NeuN, PAX6, TUJ1, MAP2 protein and glial cell markers CHAT, GFAP protein were significantly decreased (Figure 1F, Supplementary data Figure S1). These results indicate that Rotenone can significantly promote oxidative stress injury and induce death of mBOs.

### Rotenone promoted the expression of markers related to mitochondrial damage and ferroptosis in mBOs cells

**Figure 2:**
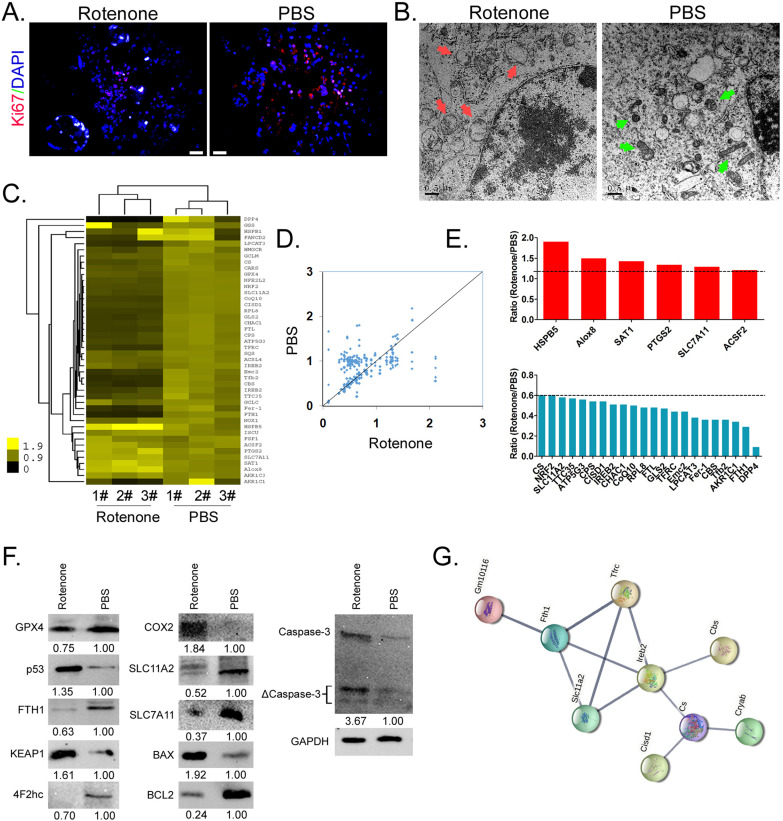
** Rotenone promoted the expression of markers related to mitochondrial damage and ferroptosis in mBOs cells. A.** Immunofluorescence staining indicated that Rotenone inhibited the expression of mBOs nuclear proliferative factor Ki67.The magnification is 200x. Scale bar = 30 µm. **B.** Transmission electron microscopy shows that Rotenone induced mitochondrial damage in mBOs. Scale bar = 0.5 µm. **C.and D.** Heat map and qPCR results showed that Rotenone treatment of mBOs resulted in differences in ferroptosis and cell cycle-related gene expression. **E.** qPCR results show that Rotenone up-regulate the expression of mBOs ferroptosis promoting gene and down-regulate the expression of Ferroptosis inhibiting gene. **F.** Westernblot results show that Rotenone significantly inhibited the expression of negative regulatory protein of ferroptosis. **G.** Protein interaction network prediction results.

Firstly, immunofluorescence staining showed that the expression level of nuclear proliferating factor Ki67 in mBOs treated by Rotenone was significantly lower than that in the control group (Figure 2A). Electron microscopy results indicated that mitochondria of mBOs in Rotenone treatment group showed swelling, deformation, vacuoles, inner ridge blur and other significant mitochondrial damage phenotypes (Figure 2B). The qPCR results showed that the expression levels of HSPB5, Alox8, PTGS2 and ACSF2, which were positively related to ferroptosis, were significantly up-regulated after Rotenone treated mBOs ([2^-ΔCt_Rotenone^/2^-ΔCt_PBS^] > 1.2). The expression levels of DPP4, FTH1, FER-1, FTL, CoQ10, NRF2 and other genes that inhibit ferroptosis were significantly down-regulated ([2^-ΔCt_Rotenone^/2^-ΔCt_PBS^] < 0.6; Figure 2C-E, Supplementary data Figure S2). At the same time, Western blot results showed that the expression of ferroptosis inhibitor protein GPX4, FTH1, 4F2hc, SLC11A2, SLC7A11 decreased significantly after mBOs was treated with Rotenone. The expression levels of ferroptosis promoting proteins p53, KEAP1, COX2, BAX and activated Caspase-3 (ΔCaspase-3) were significantly increased (Figure 2F). In addition, using the protein interaction prediction tool STING VERSION11.0 (https://string-db.org/cgi/input.pl, ©STRING Consortium 2020), we analysed signalling networks downstream of ferroptosis relative to modulating proteins. The results suggested that Ireb2 was regulated by Slc11a2, Tfrc, Fth1 and regulated downstream Cbs, Cs/Cisd1/Cryab proteins (Figure 2G, Supplementary data Figure S2). Together, these results suggest that Rotenone promotes mitochondrial damage of mBOs cells, inhibits their proliferation, activates the expression of ferroptosis promoters, and induces ferroptosis formation in mBOs cells.

### Rotenone promotes differential methylation of mBOs genomic DNA

**Figure 3:**
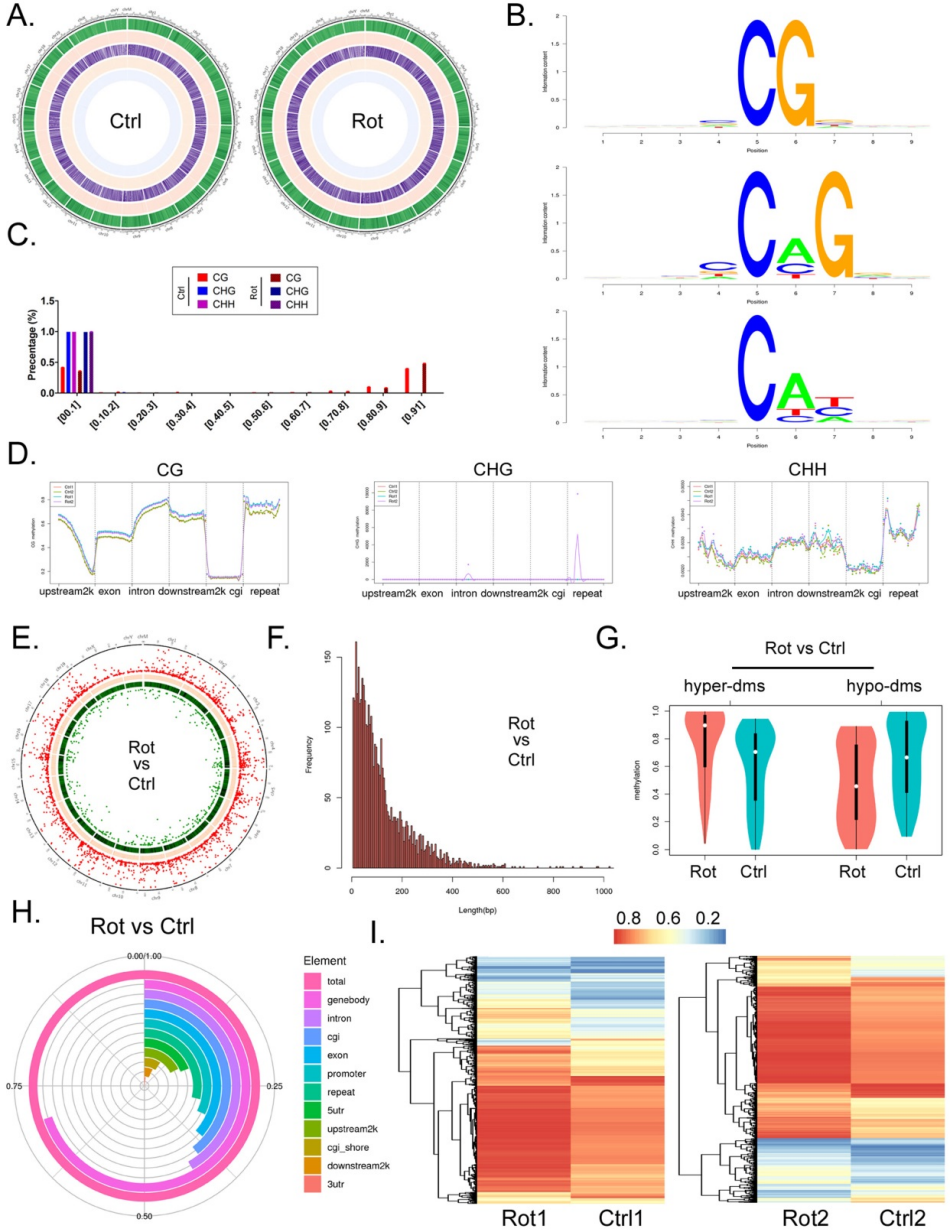
** RRBS-Seq results analysis. A.** Circos diagram shows horizontal distribution of methylation at chromosome scale.The outermost circle is the label of chromosome karyotype, and the five Heatmap bars from the outside to the inside respectively represent:GC content (Green), gene density (Red), CG methylation level (Purple), CHG methylation level (Orange), CHH methylation level (Light blue).The darker the color, the higher the level. **B.** Motif identification of methylated sites. **C.** Identification results of cytosine (C) site methylation level distribution. **D.** Comparison of methylation modification levels of motifs containing different methylation sites in functional elements of the genome among each group of samples. **E.** The Circos diagram showed the significant difference in DMR among each group. **F.** Distribution results of DMR length and frequency in each group. **G.** Statistical results of DMR methylation levels in each group. **H.** Statistical results of differential methylation level distribution in DMR anchoring region of each group. **I.** The clustering heat map showed the methylation level of DMR in each group.

We used RRBS-SEQ to analyze the differences in genomic DNA methylation modification between the two organoid groups. Sequencing results comprehensively analyzed the methylation modification differences between the two groups of samples from chromosome scale level, GC content, gene density, CG/CHG/CHH methylation level (Figure 3A). Considering that motif structural sequence of methylation site has important significance for the identification of DNA-protein binding sites, etc. We made motif seq logo for the loci information of each sequence environment (CG, CHG, CHH) and the sequence information of the identified methylated loci (9bp base including the loci) in the whole genome. The analysis results showed that the sequence characteristics of the upstream and downstream methylated cytosines of the two groups of samples in different contexts had certain commonness. The motif of CG is CG, the motif of CHG is [C/G/A]C[A/C/T]G, and the motif of CHH is C[A/T/C][T/C/A] (Figure 3B). At the same time, the results of sequencing analysis indicated that the methylation level distribution of site C on the same methylated motif in the two groups of samples was roughly the same, that is, the methylation level of CHH and CHG motif was in the range of 0-0.1% and 0.9-1%. However, the methylation level of CG motif almost occurs in a single segment of 0-0.1% (Figure 3C). In addition, methylation level analysis of functional regions of combination genes indicated that methylation level of CG motif was located in most functional elements of the genome (except downstream 2k), and rotenone-treated mbos group was slightly higher than Ctrl group (Figure 3D). However, the level of methylation modification in the motif of CHG was limited to the repeat element, and almost only the Rotenone-treated mBOs group showed high methylation level. However, the methylation level on motif of CHH was located on most genomic functional elements (except cgi), and the methylation level on repeat element was relatively high, but there was no significant difference between Rotenone-treated mBOs group and Ctrl group (Figure 3D).

Next, we analyzed the methylation differentiation levels in RRBS-Seq sequencing results differentially methylated regions (DMR). Overall analysis indicated that methylation modification at DMR level in Rotenone-treated mBOs group was significantly higher than that in Ctrl group (Figure 3E). The length involved in the two groups of DMR was approximately between 0-600 bp, and the frequency of differential methylation modification was between 150-5 (Figure 3F). Statistical results of differential methylation distribution level of DMR showed that most of THE DMR in rotenone-treated mBOs group had hypermethylation modification, while most of the DMR in Ctrl group had demethylation (low) modification (Figure 3G). The results showed that the methylation level of Genebody-DMR in Rotenone-treated mBOs group was more than 60% of the total modification level compared with Ctrl group. Secondly, promoters, Exon, Intron, CGI, repeat and other DMR are mostly located between 25% and 35% (Figure 3H). About 3860 DMR fragments were detected in the two groups of samples, and 215 highly methylated DMR modified fragments were detected (Rotenone-treated mBOs/Ctrl > 3). 142 low-methylated DMR modified fragments (Rotenone-treated mBOs/Ctrl > 3) (Figure 3I).

Therefore, the above experimental results suggest that Rotenone can indeed promote differential methylation modification of mBOs genome DNA, and the methylation modification levels of functional element regions of each genome show significant differences.

### Rotenone promotes DNA methylation and inhibits transcriptional activity of key genes in the Neuroactive Ligand-receptor interaction signal transduction pathway

**Figure 4:**
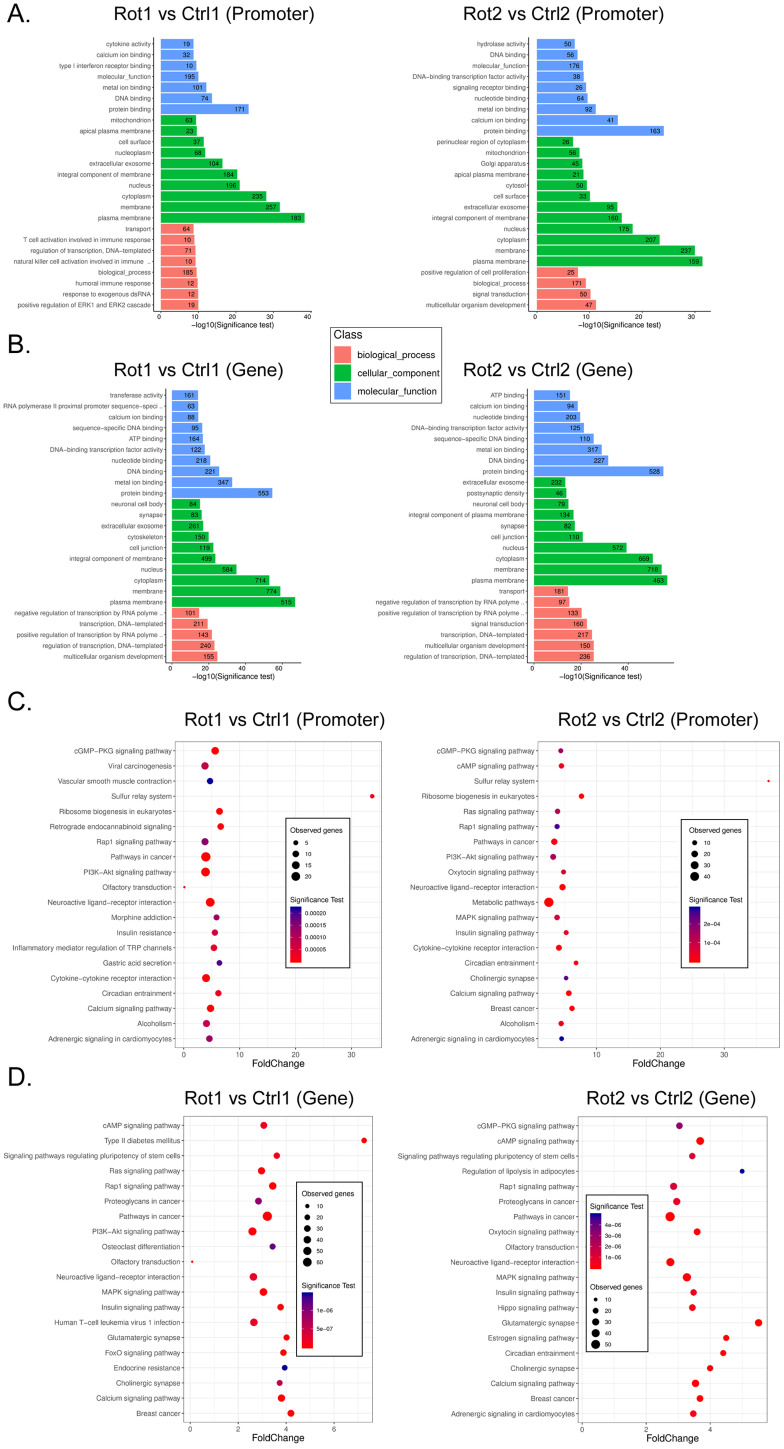
** GO and KEGG analysis results of RRBS-Seq sequencing data. A.** GO analysis revealed the functional classification population of gene promoter hypermethylation in each sample. **B.** GO analysis revealed the biological functional classification population of Genebody hypermethylation in each group of samples. **C.** KEGG analysis reveals signal transduction pathways involved in hypermethylation of gene promoters in each group of samples. **D.** KEGG analysis reveals the signal transduction pathways involved inhypermethylation of Genebody promoter in each group of samples.

It is considered that differential methylation of gene promoter and first exon plays an important role in regulating gene transcription activation. We analyzed RRBS-Seq data in detail. GO analysis showed that compared with Ctrl, rotenone-treated mBOs, the promoter hypermethylated genes were located in multicellular organism development and positive regulation of ERK1 and ERK2 Cascade, respectively biological_process classification; cellular_component plasma raft and protein binding classified by molecular_function (Figure 4A). The hypermethylated genes of Genebody are located in multicellular organism development and regulation of transcription, respectively. DNA-templated two biological_process classification; cellular_component plasma raft and protein binding classified by Molecular_Function (Figure 4B). KEGG analysis showed that compared with Ctrl, the promoter hypermethylated genes of Rotenone-treated mBOs were mainly located in the Neuroactive Ligand-receptor interaction signal transduction pathway (Figure 4C). The genes corresponding to genebody hypermethylation are located in the Neuroactive Ligand-receptor interaction, Pathways in cancer signal transduction pathway (Figure 4D).

**Figure 5:**
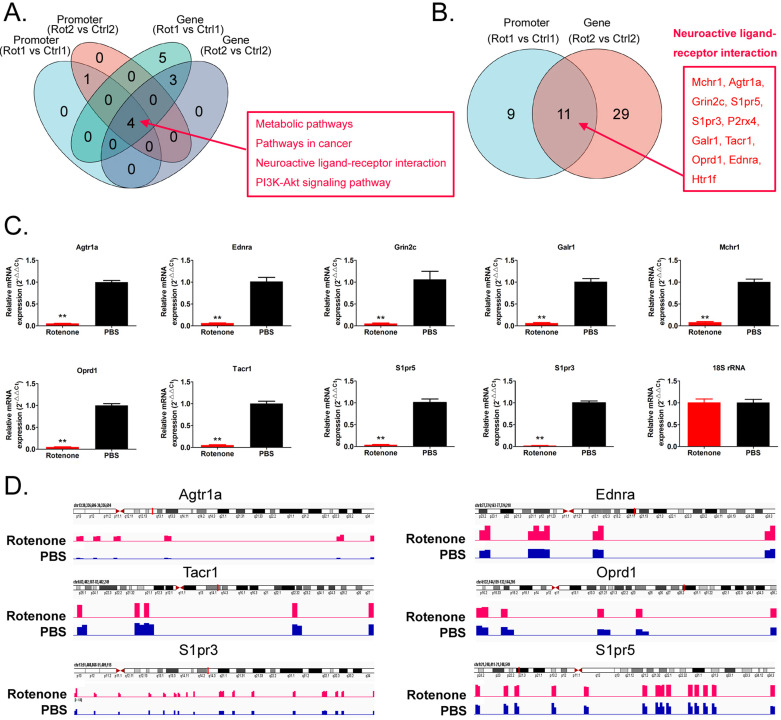
** Rotenone promotes DNA methylation and inhibits transcriptional activity of key genes in the Neuroactive Ligand-receptor interaction signal transduction pathway. A.** Venn diagram shows the signal transduction pathways involved in differential highly methylated modified genes in each group. **B.** Venn diagram shows that Promoter and Genebody are hypermethylated genes in the Neuroactive Ligand-receptor interaction signal transduction pathway. **C.** The results of qPCR detection showed that the expression level of mRNA, the key node of Neuroactive ligand-receptor interaction signal transduction pathway, in Rotenone-treated mBOs was significantly lower than that in Ctrl group.**p<0.01 vs PBS; student's t-test. **D.** The results of Integrative Genomics Viewer analysis shows that the key node genes of Neuroactive ligand-receptor interaction signal transduction pathway are all sequenced to indicate hypermethylation of promoters in Rotenone-treated mBOs.

We found that the neuroactive ligand-receptor interaction signal was transferred to the genes in the pathway, regardless of whether the promoter or the genebody was hypermethylated (Figure 5A,B). We found 11 key node genes in the above pathway. qPCR results showed that in Rotenone-treated mBOs, Agtr1a, Oprd1, Tacr1, Ednra, Grin2c, S1pr5, Galr1, S1pr3, Mchr1 mRNA expression level was significantly lower than control group (Figure 5C). And then the Integrative Genomics Viewer showed that, Agtr1a, Oprd1, Tacr1, Ednra S1pr5, S1pr3 genes have been sequenced, such as pointed out in Rotenone-treated as promoter in the mBOs and Genebody high methylation modification (Figure 5D). Therefore, experimental data suggest that Rotenone promotes DNA methylation modification and inhibits transcriptional activity of key genes in the Neuroactive Ligand-receptor interaction signal transduction pathway.

## Discussion

Rotenone has a wide range of agricultural uses. In addition to being used as an insecticide, rotenone is used as a snail killer and to catch ornamental fish 3. Rotenone was originally thought to be very safe for humans and animals, in addition to being toxic to insects and aquatic animals. However, more and more studies have reported that rotenone has high toxicity to domestic animals, fish, silkworm and mice, especially neurotoxicity and reproductive toxicity 4-8. Moreover, with the widespread use of rotenone, it has gradually become a hidden ecological toxin and environmental pollutant. The initial study found that when rotenone entered the insect body, it would inhibit the mitochondrial electronic respiratory chain of insect somatic cells, leading to respiratory disorders such as dyspnea and convulsion, slow movement, paralysis and death 2. However, with the deepening of neurotoxicological research, more and more reports confirm that the toxicological mechanism of rotenone is far from simple and simple. Our previous study indicated that rotenone can affect covalent modification of mouse dopamine neuron histones and transcriptional activity of target genes 9, these results suggest that rotenone has epigenetic toxicity. However, in-depth systematic research data have not been reported.

Firstly, in this study, we want to explore whether rotenone has the potential to induce other types of death in addition to neuron necrosis. Considering that rotenone can cause oxidative phosphorylation (electron respiratory chain transmission) abnormalities, we targeted ferroptosis. As electron microscopy results showed that Rotenone promoted mitochondrial damage in mBOs cells, and biochemical tests also suggested that Rotenone promoted significant increases in lipid peroxides and Fe^2+^ in mBOs, we speculated that rotenone-induced death of mBOs belonged to the category of ferroptosis. Ferroptosis is a new type of iron-dependent programmed cell death, which is different from apoptosis, cell necrosis and autophagy 42-47. The main mechanism of ferroptosis is that under the action of divalent iron or ester oxygenase, the unsaturated fatty acids with high expression on the cell membrane are catalyzed to produce liposome peroxidation, thus inducing cell death. In addition, the expression of GPX4 in antioxidant system (lipid peroxides) was decreased 42-47. GPX4 is the only glutathione peroxidase (GPX) used for liposome peroxides 47. GPX4 can transform the peroxide bond of lipid peroxidation into hydroxyl group and lose its peroxide activity. It is found that when the iron ions are injected into the cells and the iron ions exist in the form of ferrous iron, when the iron overload state is reached, the liposome peroxidation can be started. Peroxidase liposome can significantly digest the activity of GPX4 *in vivo* and induce ferroptosis 47. When ferroptosis occurs, the mitochondria of the cells become smaller, the membrane density increases, and the cristae decreases, accompanied by an increase in lipid peroxidation and ROS in the cytoplasm. At present, it has been reported that Ferroptosis can significantly promote the death of cancer cells and inhibit their activities of proliferation, division and invasion. However, it is not clear whether Rotenone will induce the death of mBOs iron. We further confirmed that after Rotenone treatment of mBOs, the expression of ferroptosis inhibitory proteins such as Gpx4, FTH1 was significantly decreased, while the expression of ferroptosis promoting proteins such as COX2, P53 and KEAP1 was significantly increased. Therefore, we have reason to believe that Rotenone has a potential molecular biological basis for inducing ferroptosis in mBOs.

Once the phenotype is clear, our next task is to reveal the molecular mechanism by which rotenone induces mBOs ferroptosis. Since our previous studies focused on epigenetics, we are still interpreting the underlying mechanisms behind phenotypes from an epigenetic perspective. Considering that DNA methylation modification (an important part of epigenetics) has a very important impact on gene transcription and even expression activity, DNA hypermethylation can silence gene transcription and expression. We used simple genomic DNA methylation high-throughput sequencing to explore which gene methylation differences were caused by rotenone and which functional element regions of the genome the differences occurred in. The results showed that Rotenone could induce hypermethylation of mBOs, and the methylation level of genebody-DMR accounted for more than 60% of the total methylation level. It can be seen that rotenone mainly stimulates hypermethylation of CpG island in genebody, followed by Promoter region. Through bioinformatics analysis, it is speculated that most genes located in the Neuroactive ligand-receptor interaction signal transduction pathway will undergo hypermethylation in promoter and gene-body dual functional regions. Since neuroactive ligand-receptor interaction is a signal pathway controlling the neuron to receive and release impulses, the inactivation of this pathway will directly lead to the neuron unable to respond to external stimuli normally. However, none of the above studies have been reported in the current study on the toxicological effects of rotenone.

**Figure 6:**
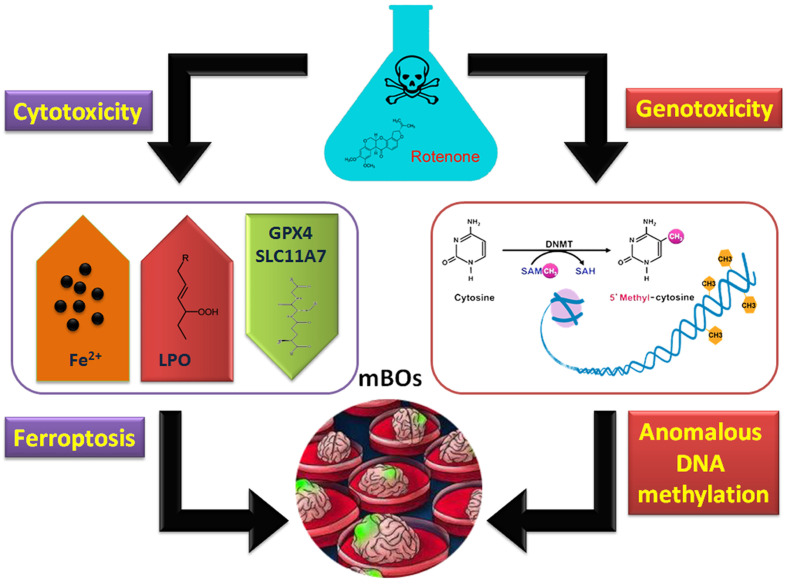
Molecular mechanism of abnormal methylation of the organoid genome and ferroptosis induced by rotenone.

In conclusion, rotenone can induce ferroptosis in brain organs of mice. On the other hand, it has genetic toxicity that activates abnormal methylation modifications in the genome (Figure 6).

## Supplementary Material

Supplementary figures.

## Abbreviations

mBOs: Brain organs
LPO: Lipid peroxides
GO: Gene Ontology
KEGG: Kyoto Encyclopedia of Genes and Genomes
Ctrl: Control group
MDA: Lipid Peroxidation
TBA: Thiobarbituric acid
H&E: Hematoxylin and eosin
RRBS-Seq: High throughput sequencing of genomic DNA methylation sequencing
DMR: Differentially methylated region
