# Supplementary material for: Rotenone, an environmental toxin, causes abnormal methylation of the mouse brain organoid's genome and ferroptosis
Source: Int J Med Sci. 2022 Jul 4;19(7):1184–97. doi: 10.7150/ijms.74569 (PMC9339416; doi:10.7150/ijms.74569)
Supplement: Supplementary file 1 — Supplementary figures. [file ijmsv19p1184s1.pdf]

Rotenone, an environmental toxin, causes abnormal methylation of the mouse brain organoid's genome and ferroptosis

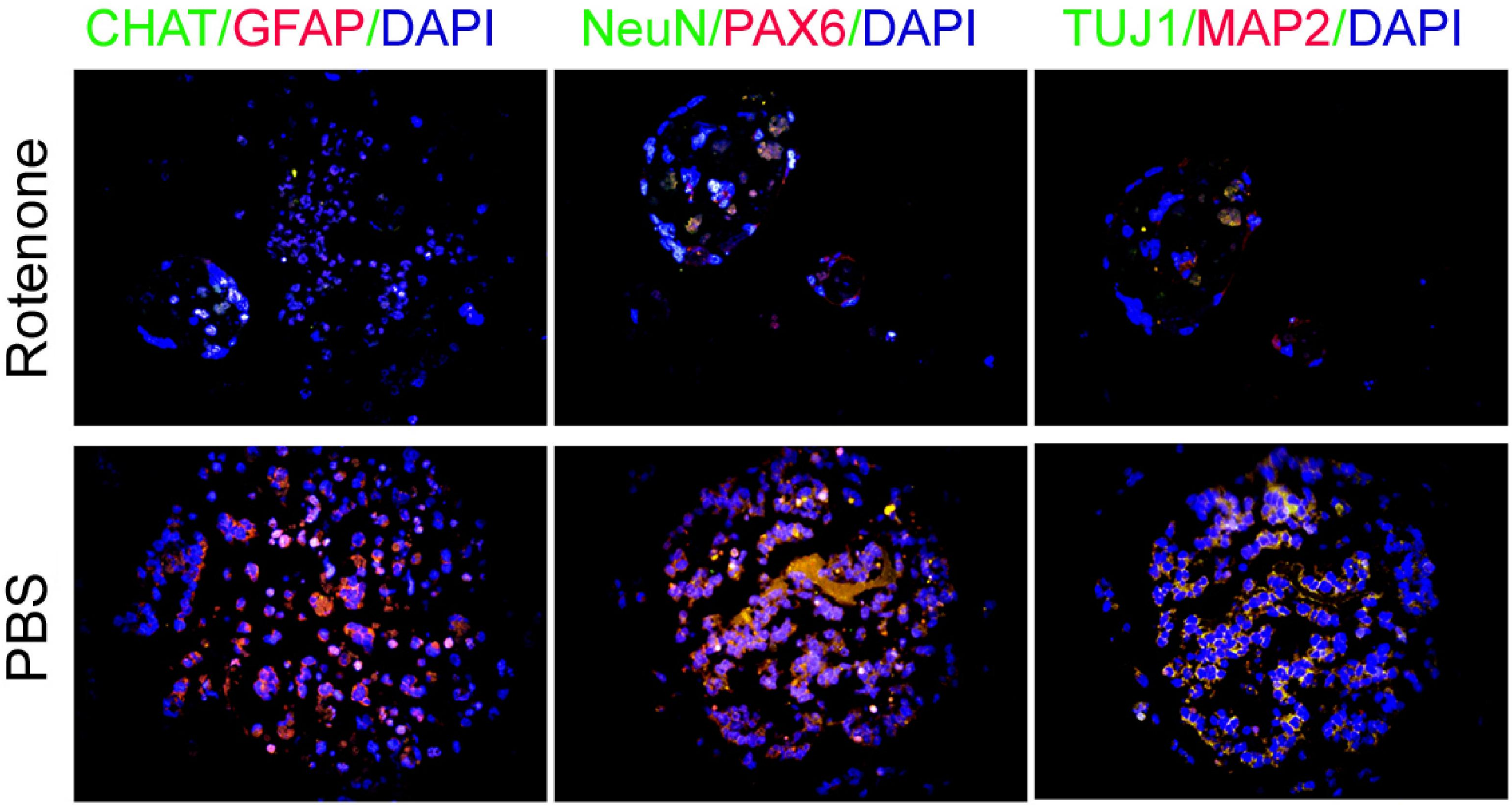

Figure S1 Immunofluorescence staining indicated that Rotenone inhibited the expression of neuronal markers in NE-4C-derived mBOs.The magnification is 200x.

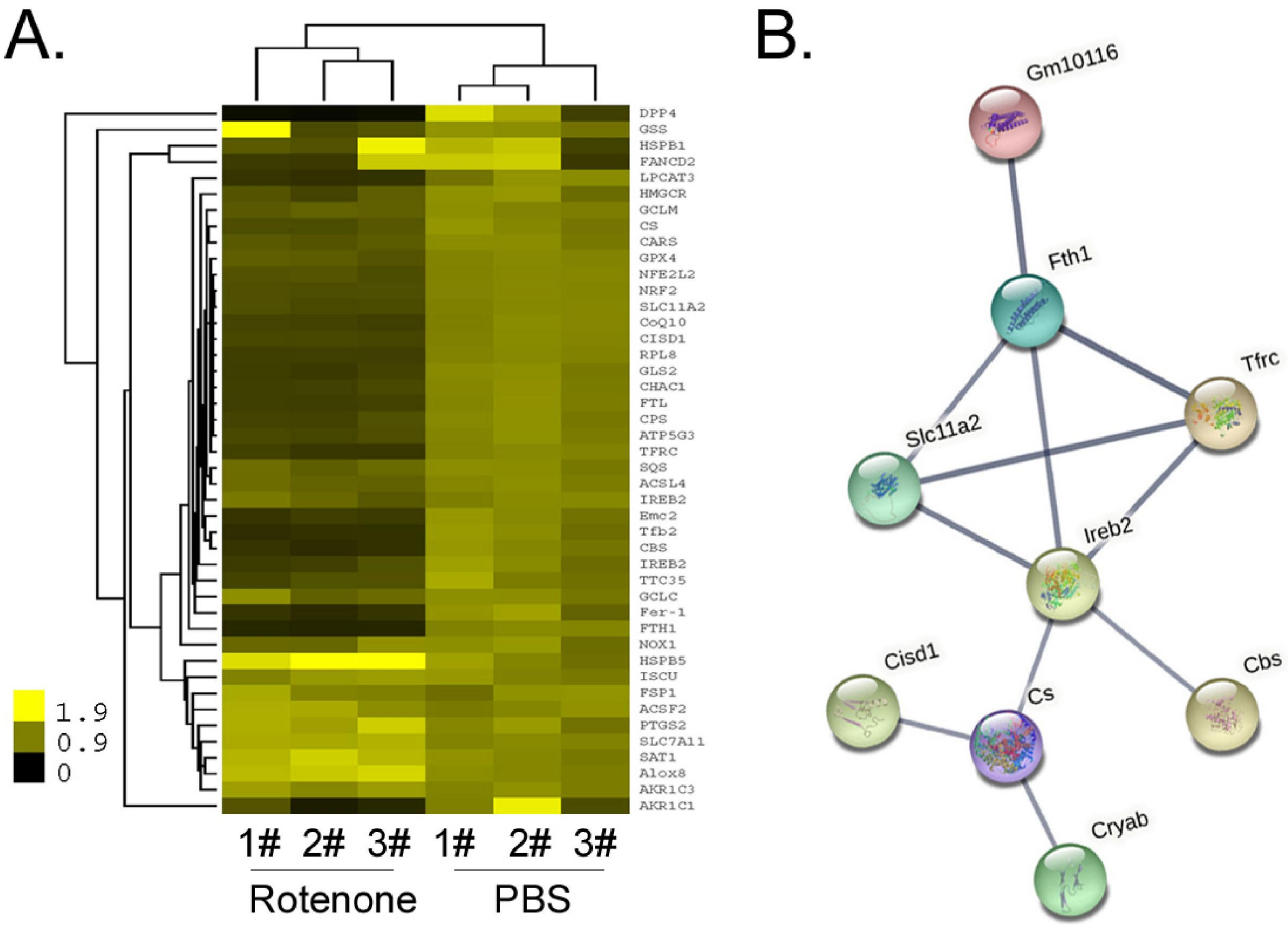

Figure S2 Rotenone promoted the expression of markers related to mitochondrial damage and ferroptosis in mBOs cells

A. Heat map results showed that Rotenone treatment of mBOs resulted in differences in ferroptosis and cell cycle-related gene expression.

B. Protein interaction network (PPI) prediction results.
